# Quantifying developmental and individual differences in spontaneous drawing completion among children

**DOI:** 10.3389/fpsyg.2022.783446

**Published:** 2022-11-11

**Authors:** Anja Philippsen, Sho Tsuji, Yukie Nagai

**Affiliations:** ^1^International Research Center for Neurointelligence (WPI-IRCN), The University of Tokyo, Tokyo, Japan; ^2^Institute for AI and Beyond, The University of Tokyo, Tokyo, Japan

**Keywords:** representational drawing, cognitive development, autistic traits, convolutional neural network (CNN), individual differences, predictive coding

## Abstract

This study investigated how children's drawings can provide insights into their cognitive development. It can be challenging to quantify the diversity of children's drawings across their developmental stages as well as between individuals. This study observed children's representational drawing ability by conducting a completion task where children could freely draw on partially drawn objects, and quantitatively analyzed differences in children's drawing tendencies across age and between individuals. First, we conducted preregistered analyses, based on crowd-sourced adult ratings, to investigate the differences of drawing style with the age and autistic traits of the children, where the latter was inspired by reports of atypical drawing among children with autism spectrum disorder (ASD). Additionally, the drawings were quantified using feature representations extracted with a deep convolutional neural network (CNN), which allowed an analysis of the drawings at different perceptual levels (i.e., local or global). Findings revealed a decrease in scribbling and an increase in completion behavior with increasing age. However, no correlation between drawing behavior and autistic traits was found. The network analysis demonstrated that older children adapted to the presented stimuli in a more adult-like manner than younger children. Furthermore, ways to quantify individual differences in how children adapt to the presented stimuli are explored. Based on the predictive coding theory as a unified theory of how perception and behavior might emerge from integrating sensations and predictions, we suggest that our analyses may open up new possibilities for investigating children's cognitive development.

## 1. Introduction

A way to naturally gain insight into children's cognitive development is by observing their spontaneous drawings (Thomas and Silk, 1990; Adi-Japha et al., 2010; Pace et al., 2022a). Drawing ability makes for an appealing study subject as it is a complex task that involves cognitive as well as motor and perceptual skills, yet producing a structured, relatively simple, two-dimensional outcome: lines on a piece of paper or a drawing device. Over many decades of research, it has been shown that children's drawings reflect their developmental maturation (Thomas and Silk, 1990; Adi-Japha et al., 2010; Saito et al., 2014) and can help assess how children perceive their environment (Chappell and Steitz, 1993; Barraza, 1999). Drawing has also been suggested to provide insights into children's development that can be considered to be relatively independent of the linguistic and cultural background of a child (Pace et al., 2022a,b). Of particular interest is the development of representational drawing ability that children typically acquire within the first few years of their life: they start drawing objects or people instead of meaningless scribbles. Research on the development of representational drawing ability (Selfe, 1983; Yamagata, 2001; Ford and Rees, 2008; Saito et al., 2014) can help us understand how children develop abstract thinking and make the connection between a real object and its representation in a picture, which are fundamental abilities in human cognition.

However, a central challenge in the study of children's drawing ability is the interpretation of produced drawings. Past attempts have been mainly based on relatively small sample sizes, and the drawings have been primarily assessed based on the subjective judgments of a small number of people (Yamagata, 1997, 2001; Adi-Japha et al., 2010; Thomas et al., 2010; Saito et al., 2014). New methodological and computational advances that have emerged in the last few decades could help address this issue. Specifically, drawing data can be recorded directly using electronic devices such as tablet PCs, and crowdsourcing platforms and computational methods could enable quantitative data analysis (Paolacci et al., 2010; Long et al., 2018). The present study aimed to apply such new methodologies for studying children's drawings, with the goal of quantifying the developmental and individual differences among them.

As a theoretical framework of our analyses, we use the predictive coding theory (Rao and Ballard, 1999; Friston, 2009; Ciria et al., 2021). This theory explains the mechanisms underlying perception and behavior based on the idea that sensory signals (bottom-up information) are integrated with prior predictions (top-down information) in order to constantly make predictions about the world. Making predictions is a fundamental mechanism that underlies various cognitive functions and plays an important role in development (Trainor, 2012; Ylinen et al., 2017; Nagai, 2019). In predictive coding, the precision of sensory and prior information affects perception and behavior: individuals rely more on precise signals compared to those which are imprecise. A stronger or weaker reliance on either sensory input or predictions is discussed as a potential reason for divergence in perception and behavior between different age groups or across developmental conditions (Gonzalez-Gadea et al., 2015; Sterzer et al., 2018; Lanillos et al., 2020). Therefore, the framework of predictive coding could provide a mechanistic explanation of developmental and individual differences in children's behavioral tendencies.

To investigate children's drawing skills in the context of predictive coding, one type of drawing task that appears particularly suitable is drawing completion. While it was originally introduced at the beginning of the twentieth century to test children's cognitive abilities (Pintner and Toops, 1918; Ames, 1945), a spontaneous drawing completion task was recently used to investigate the emergence of representational drawing ability in a systematic manner (Saito et al., 2014). In their study, Saito and colleagues presented incomplete drawings to children between 6 and 38 months of age, where essential parts of the drawing, including facial features such as eyes or the mouth, were missing. It was observed that children showed a tendency to draw in those regions where parts were missing, which could be interpreted as a tendency to complete drawings. Thus, children from the age of 2–3 years, although possessing motor skills that need further maturation, already developed the cognitive processes required to associate an incomplete drawing with a concept.

The drawing completion task can be considered as a prediction task: in order to decide what to draw, children have to integrate the perceived sensory information (bottom-up information) with their priors (top-down information). Priors may include knowledge about concepts, which are needed to successfully complete a drawing, but can also include personal preferences. For example, a child might prefer to draw a specific object, regardless of the presented shape, indicating an influence mainly by top-down information. In contrast, if bottom-up information had a stronger affect, they would keep close to the presented drawing. Successful completion of the drawing can be expected only if top-down and bottom-up information are adequately integrated.

Unlike general drawing tasks, drawing completion is also useful for a systematic analysis since the task limits the potential behaviors that the child might show. Another advantage is that it does not require instructions, thus, drawing completion makes it possible to investigate the drawing ability of very young children or children with linguistic deficits, for instance, in the context of developmental disorders.

Therefore, in this study, an extended version of the drawing completion task of Saito et al. (2014) was designed. Specifically, the task was adapted to investigate drawing within the predictive coding framework, by introducing a larger variety of stimuli designed to provide drawings of different object categories (activating different top-down concepts), along with different ways to present these drawings (modifying bottom-up information).

In order to examine individual differences, we measure children's autistic traits (AQ score, Auyeung et al., 2008). The reason is that predictive coding has been commonly applied to investigating behavioral differences in developmental conditions such as autism spectrum disorder (ASD) (Lawson et al., 2014; Van de Cruys et al., 2014; Sterzer et al., 2018). In the context of drawing, atypical drawing has been widely observed in the context of ASD, with some children with ASD showing highly realistic drawing ability at a very young age (Selfe, 1983; Snyder and Thomas, 1997; Cox and Eames, 1999). However, children with ASD generally seem to perform worse than typically developing children in drawing tasks (Fuentes et al., 2009; Kushki et al., 2011; Fleury et al., 2013; Johnson et al., 2015). Autistic traits, as measured by this questionnaire, are also widespread in the general population, and have been shown to correlate with differences in perception (Reed et al., 2011; Tavassoli et al., 2014; Ujiie et al., 2015). We hypothesized that these individual differences, measured as AQ scores, might account for some of the variability observed in children's drawings.

The current study applied improved methodologies by collecting a drawing set from a larger sample size (621 drawings from 114 children) compared to previous studies, and by applying an objective data analysis process. Specifically, the analysis entailed two approaches for evaluating children's drawing data, that could be easily scaled to large datasets. First, a crowdsourcing study was performed to collect ratings from adults about features that are visible in the drawings. Second, the drawings were evaluated based on a computational technique that used visual features extracted from deep convolutional neural networks (CNNs). This technique was recently proposed by Long et al. (2018) for examining children's drawings at different levels of abstraction, spanning from local to their more global features. Specifically, we investigated whether the technique is suitable for measuring developmental and individual differences in children's drawings.

The results presented in this paper are an extension of a previously published study (Philippsen et al., 2020), including additionally an analysis and discussion of children's autistic traits along with an exploratory investigation of individual differences that were found in children's drawings.

## 2. Methods

The experiment was conducted in a science museum in Tokyo (Miraikan Science Museum) between October 2019 and February 2020, in a separate area where children with their parents could enter free of charge. Participants were recruited on the day, or could register in advance. The parents were informed of the content of the experiment, and informed consent was obtained from all participants. The experiment was reviewed and approved in advance by the research ethics committee of the Office for Life Science Research Ethics and Safety at The University of Tokyo to confirmed that the study conforms to recognized standards.

### 2.1. Experiment design

**Figure 1:**
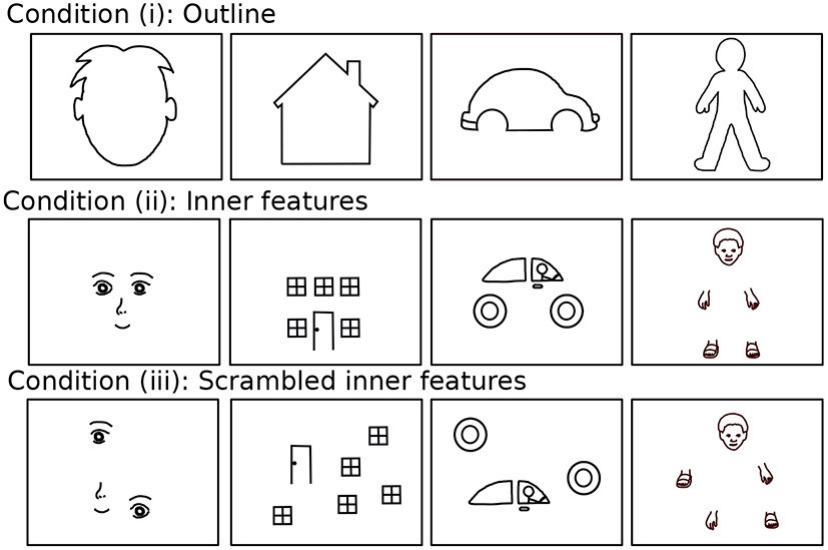
The twelve stimuli of which six were presented to each of the children, consisting of four different stimuli categories (face, house, car, and human figure) and three presentation conditions (outline, inner features, scrambled inner features). Reproduced with permission from Philippsen et al. (2020).

The experiment was designed as a completion task (Saito et al., 2014). While the original study only used one type of stimulus, namely a face with some missing inner features (e.g., a missing eye), the present research aimed to design a more diverse set of stimuli by constructing varying object categories and configurations, as shown in Figure 1. Four different object categories (face and human figure as animate objects, and house and car as inanimate objects) were selected such that they all contained features that are indispensable for defining a typical example of the category (e.g., eyes are essential for a face, and wheels for a car). In addition, we presented these stimuli in three different configurations. (i) In the *outline condition*, the outline of the object was shown without any detailed features. Sketching the outline could be the most typical way of getting started when drawing an object (Booth et al., 2003); thus, presenting the outline could prompt children in a natural way to add inner features to the drawing. (ii) The inner parts of the drawing, such as the eyes and mouth of the face, are shown in the *inner feature condition*. In order to complete a drawing of this nature, by connecting the presented parts or adding the missing outline, the child needs to recognize the parts and make sense of their configuration. (iii) In the *scrambled inner feature condition*, the features from condition (ii) were presented in a scrambled configuration, such that the typical completion of the object was not possible. Without a straightforward solution, this condition is more difficult than (i) and (ii), as the presented objects had to be reinterpreted or combined to achieve proper completion. In addition, this condition may elucidate individual differences related to autistic traits (see the hypotheses listed in the Section 1).

Importantly, the three presentation conditions for a single object differed visually from each other. They provided different bottom-up information to the child, but semantically pointed to the same object, thus, activating the same top-down concept. This feature of the stimuli set later enabled an investigation of how changes in the presented sensory stimuli affect children's drawing tendencies (cf. Section 4).

Each child drew up to six of the available stimuli; specifically, the child was assigned (in order of arrival) to one of four previously prepared drawing sets that contained six drawings each (two of each of the three presentation conditions, containing a maximum of two times the same pattern category).

Children drew directly on a tablet PC to allow for an automated analysis.

### 2.2. Experiment procedure

**Figure 2:**
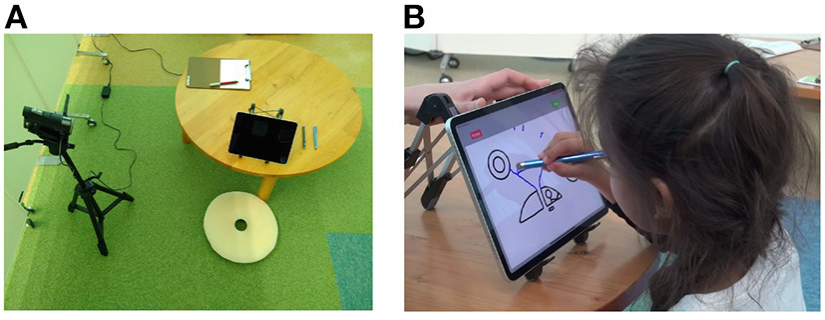
Setup of the experiment in the museum. **(A)** View on the tablet PC used for drawing, the child sits in front, the experimenter on the left side of the table. **(B)** A child drawing on one stimulus.

The experiment was performed in a separate area and children sat with the back to the entrance to keep distractions to a minimum; however, since it was located in an open space connected to the rest of the museum, ambient noise could not be avoided. Whenever possible, children performed the task alone, guided only by the experimenter; younger children could sit on their caretaker's lap while drawing. The children were presented with pictures on a tablet PC (IPad Pro 11 inch) and were told to draw freely on the stimuli. A blue stylus pen was provided for drawing. The original drawing was shown in black on the tablet PC. The children's drawings were displayed in blue to enable differentiation from the original drawing.The experimental setup is shown in Figure 2.

During or immediately after the experiment, parents were asked to complete the child version of the AQ score questionnaire (Auyeung et al., 2008), which consists of 50 statements about the child's social and communicative skills, imaginative ability, attention switching ability, and attention to detail. Parents were asked to rate whether the statements applied or not, and each statement was scored as 1 if children show more autistic-like behavior and 0 otherwise. The resulting AQ score measured how pronounced the autistic traits of the child were.

### 2.3. Participants

Individuals of all nationalities could participate, but the majority were Japanese. A total of 114 children between 2 and 8 years of age participated in the study. Ten of them had to be excluded based on the criteria for exclusion that are stated in the preregistration. Specifically, four children (one 4-year-old, one 6-year-old, and two children of unknown age) were excluded because they ran away from the experiment before drawing on at least one picture per condition. Two children (5 and 6 years old) were excluded because they only drew something on the free trial drawing in the beginning but did not draw anything on any of the stimuli. Three children had to be excluded due to technical issues with the iPad (4 and 5 years old) or due to problems with handling the iPad (4 years old). One child (4 years old) was excluded because the caregiver helped the child by verbally hinting at what she should draw albeit instructed otherwise.

The remaining 104 children (62 males and 42 females) produced a total of 621 completion drawings that were included in the analysis. The average age of these children was 4 years and 9 months (range, 2 years and 1 month to 8 years and 3 months), with a standard deviation of 16 months^1^.

As participation was spontaneous, not all participants were able to stay long enough to complete the AQ score questionnaire. In addition, the AQ score questionnaire is designed for children of ages 4 years and above, thus posing some difficulties for parents of younger children to accurately respond. In total, only 65 questionnaires among the 104 children were filled completely; however, in most cases, only a few questions remained unanswered. Therefore, the AQ score was used for analysis if the parents filled in at least 80% of the AQ score questionnaire, that is, if they answered a minimum of 40 of the 50 questions. If less than 50 questions were answered, the resulting score was scaled accordingly to be equivalent to a fully filled questionnaire. Thus, the AQ scores of 94 children could be included in the analysis. While the age and AQ score of the children were well-distributed, approximately 60% of the children had an AQ score between 10 and 20. Only six children had an AQ score of above 32, which is usually applied as a cut-off indicating strong autistic tendencies (Auyeung et al., 2008). However, it is important to note that the score does not necessarily suggest an ASD diagnosis (i.e., a considerable number of children would score higher than 32 while developing typically).

To account for the fact that the AQ score questionnaire is designed for children of 4 years or older, we repeated the analyses related to the AQ score with a subset of children of 4 years and older, as detailed in the preregistration. The results reported here also hold for the subset analyses, thus, we do not report the analyses in detail.

To enable comparisons between child and adult drawings for similar stimuli, an additional 107 drawings were collected from five adults who could draw up to two times on each of the stimuli.

### 2.4. Evaluation

Two complementary methods were used to analyze the data. First, an adult rating study was performed using crowdsourcing (Section 2.4.1) to investigate the drawing tendencies found in the collected data set. This analysis aimed to categorize children's drawing behavior with greater reliability than that achievable by current computational methods. For instance, in order to decide whether a drawing was completed or not, it is important to use contextual knowledge of what the drawing should represent and whether the child's drawing is meaningful within the context of the presented stimulus. Additionally, the human rating provides a baseline that can be used to confirm the validity of the subsequent computational analysis.

Second, the data was evaluated based on visual features extracted from the raw drawings following the methodology proposed by Long et al. (2018). In contrast to the rating analysis which required predefined rating categories, this analysis enables a more objective evaluation of the drawings. Specifically, a pretrained deep CNN (Section 2.4.2) was used to analyze the drawings at different layers of visual processing (local vs. global) and to investigate how children adapted their drawings in response to a change in the presented stimulus.

#### 2.4.1. Adult rating study

The adult rating study was conducted using Amazon Mechanical Turk, and was designed to verify our hypotheses about differences in the drawing style of the children (see Section 2.5). Recruited participants were presented with one of the children's drawings and asked to rate whether statements about the particular drawing were false or true, using a continuous scale ranging from 0 (false) to 100 (true).

As a behavioral measure of drawing, we were primarily interested in the general features of the produced drawing rather than in its semantic content. Here, we use the term *drawing style* to refer to the broad features of drawings; specifically, we consider “completion,” “scribbling,” “coloring in” of shapes, and the “tracing” of existing lines, inspired by drawing behaviors that were reported in Saito et al. (2014). Accordingly, in the rating study, participants were asked to rate the drawing based on seven different statements. For each item, raters could adjust a slider between 0 and 100 to indicate whether the statement applied to a drawing. For completion, the following statements were used:

- “The child completed the drawing by adding an outline.”
- “The child completed the drawing by adding parts to it.”

For scribbling, the following statements were used:

- “The child scribbled randomly on the original drawing.”
- “The child scribbled randomly on blank space.”

From the responses, a *completion score* and a *scribbling score* were computed by averaging the related questions.

Additionally, statements pertaining to alternative drawing styles such as coloring in or tracing were asked:

- “The child traced/redrew or copied existing lines.”
- “The child colored in (parts of) the drawing.”

Finally, a general question that could provide a hint of whether the drawings of the children were semantically linked to the presented part was added:

- “What the child drew (in blue) is related to the original drawing (black).”

In the instructions, examples of the intended drawing behavior were shown to ensure that the participants understood the intended meaning of the statements. These examples were selected from images that were rated with the maximum score in the corresponding question, from a pilot rating which contained the drawings of the first 27 children.

The ratings for a total of 570 pictures were obtained online. Five independent ratings were obtained for each of the drawings. Raters could rate as many different drawings as they liked. A total of 417 raters participated in the study, each of whom rated an average of six drawings. For four children, the parents did not provide consent for anonymous use of their children's drawings on online platforms. Therefore, the 24 drawings of these children were separately rated offline by four adults who were not involved in this research project. Children's drawings in which nothing was drawn (27 out of the 621 obtained drawings) were automatically assigned 0 for all statements.

As a known-answer question, rating study participants were additionally asked what they thought the black part of the drawing represented (choices were “face,” “house,” “car,” “human figure,” or “I do not know”). The ratings provided by a participant were included in the subsequent analysis only if the participant answered correctly in more than 70% of the ratings.

For statistically testing the hypotheses, we use linear mixed-effect models for regression modeling and the chi-squared test (likelihood-ratio test) to compare the goodness of fit of a model with vs. without a variable in question (e.g., age) to determine statistical significance of that variable.

#### 2.4.2. Convolutional neural network analysis

**Figure 3:**
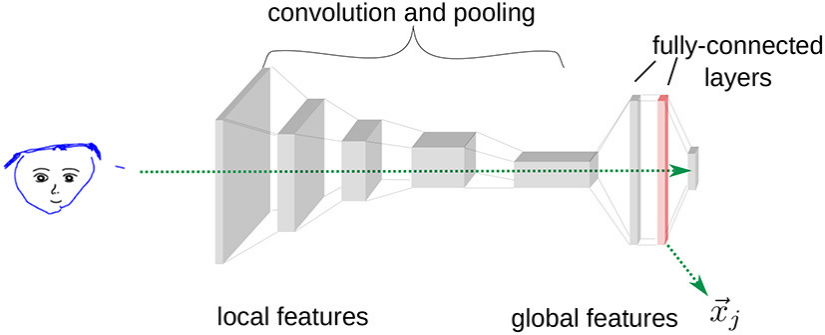
The structure of the pretrained deep CNN of Simonyan and Zisserman (2014), consisting of five conjunctions of convolution and pooling layers, two fully connected layers, and the classification output layer. The input image is passed through the layers, and local or global features of the image can be extracted at early or later layers, respectively (as exemplified in the second fully connected layer).

The human rating study conducted here is based on predefined rating statements. Therefore, analyzing the drawings based on computational image analysis techniques could be useful to provide an efficient and objective analysis at either the local or global level. For the analysis of children's drawings, Long et al. (2018, 2019) proposed the use of a deep CNN, pretrained for a large dataset of objects and scenes. A CNN is a type of neural network that is commonly used for image and scene classification (Rawat and Wang, 2017) and structured in a hierarchical order inspired by the visual cortex of the human brain (Cadieu et al., 2014). Specifically, CNNs consist of a cascade of layers that gradually perform feature extraction on the previous layer's result via convolution with adaptable filters and downsampling of the resulting features (pooling), as shown in Figure 3. During training, the weights of the filters are adapted such that features are extracted that are useful for differentiating the training data. The hierarchical nature of the CNN permits an analysis of the children's drawings at different levels: the lower layers of the CNN extract more local, low-level features of the image, such as the orientation of edges, and the higher layers of the CNN extract more global, high-level features that relate more strongly to the conceptual meaning of the image (Cadieu et al., 2014). Using the CNN features, it becomes possible to directly compare the features of two arbitrary drawings in order to measure the similarity between them.

Note that in the study of Long et al. (2019), the CNN is used to evaluate the recognizability of the produced drawings as distinct object categories, making it necessary to train an additional classifier. In contrast, in the present study, we are not only interested in representational drawing but also in other drawing styles. Therefore, we use the CNN features directly to measure *relative* differences between drawing styles, for instance, for comparing the drawings of adults and of children, or the drawings created for different conditions.

Here, the VGG19 network architecture proposed by Simonyan and Zisserman (2014) was used, which was pretrained on the ImageNet dataset (Deng et al., 2009), containing millions of labeled photographs of objects and scenes. Figure 3 shows a simplified sketch of the CNN used, which consists of five conjunctions of a convolutional and a pooling layer, two fully connected layers, and a classification output layer. To extract the global features of a child's drawing, it is passed through the network and the activations of an intermediate layer are used as features of that drawing. Networks trained on this type of dataset have been previously applied primarily to photographic images; however, it has been shown that the extracted features also transfer to other types of images, such as medical data (Shin et al., 2016). Here, a verification analysis was conducted to ensure that the CNN is capable of extracting useful features of line drawings (see Section 4.1).

The procedure in Long et al. (2018) was followed: all drawings were resized to 224 × 224 pixels and preprocessed according to Simonyan and Zisserman (2014) before feeding them into the network. The features obtained from the network were used for two different types of comparisons. First, a comparison was made of how two different individuals drew on the same presented stimulus. The full drawing, including the presented and completed parts, were used in this case because the presented parts of both drawings were identical. Second, the features were used to compare how one child drew on one stimulus in comparison to another stimulus. In this case, the presented part of the drawing was omitted, and only the completed part was used for the analysis. In both cases, the blue parts of the image were changed to black to eliminate the differentiation between the presented and completed parts, which is irrelevant for the CNN analysis.

As a measure of dissimilarity between different feature vectors, the Pearson correlation was utilized in accordance with Kriegeskorte et al. (2008) and Long et al. (2018). Formally, the distance (or dissimilarity) $d\left({\vec{x}}_{i},{\vec{x}}_{j}\right)$ between two feature vectors were defined as:


(1) $$\begin{array}{l} d\left({\vec{x}}_{i},{\vec{x}}_{j}\right)=1-\frac{\text{cov}\left({\vec{x}}_{i},{\vec{x}}_{j}\right)}{\sqrt{\text{var}\left({\vec{x}}_{i}\right)\cdot \text{var}\left(\vec{x_{j}}\right)}}, \end{array}$$

where ${\vec{x}}_{i}$ and ${\vec{x}}_{j}$ were two vectors corresponding to the neural activations in one of the network layers when presenting images *i* and *j* as inputs.

### 2.5. Hypotheses

Five preregistered hypotheses^2^ were tested based on the crowdsourcing analysis. The first three hypotheses were related to the children's development. Based on literature on children's drawing skills (Saito et al., 2014), it was expected that with increasing age, children would complete more and scribble less, across all object categories and presentation conditions:

1. With increasing age, children exhibit more representational drawings (i.e., complete the stimuli more frequently).
2. With increasing age, children exhibit less scribbling.
3. Children draw in a similar manner, regardless of whether the outline [condition (i)] or the inner parts are presented [condition (ii)].

Furthermore, different tendencies in developmental improvement were expected in children with stronger autistic traits. Differences in drawing related to autistic traits may arise from various factors. For example, a local processing bias has been found in individuals with ASD (Mottron et al., 2003; Behrmann et al., 2006), which appears to cause a higher fragmented drawing tendency, leading to a focus on local features or violation of the configurations of a drawing (Booth et al., 2003). More recently, it has been suggested that individuals with ASD might have a weaker tendency to use prior predictions when processing sensory input (Pellicano and Burr, 2012), which could also result in difficulties with completing representational drawings (Philippsen and Nagai, 2019). Therefore, an overall lower rate of completion with higher AQ scores was expected. Furthermore, the difficulties with using prior predictions and the tendency to violate configurations led to the prediction that children with a lower AQ score would more strongly adjust their drawing to the scrambled vs. non-scrambled conditions, responding with different types of drawing behavior, whereas children with a higher AQ score might draw similarly in both conditions. These expectations are expressed by these two hypotheses:

4. Children with a higher AQ score are less likely to perform representational drawings.
5. Children with a higher AQ score draw in a more similar manner when comparing the inner features [condition (ii)] and the scrambled inner features conditions [condition (iii)].

## 3. Results I: Adult rating study

**Figure 4:**
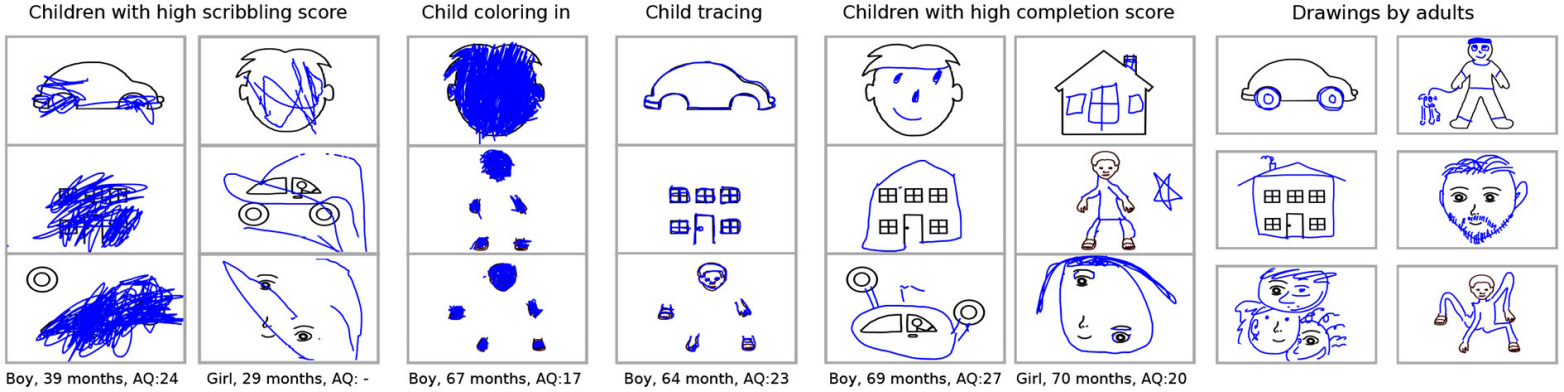
Examples of drawings of children who received particularly high ratings for different rating categories, and of adult's drawings.

As described in Section 2.4.1, adult ratings for the drawings were obtained to investigate which drawing styles children showed at different ages. Figure 4 shows examples of drawings of those children who achieved particularly high ratings for the four drawing style categories (scribbling, coloring, tracing, and completion), along with comparison drawings that were created by adults on the right.

**Figure 5:**
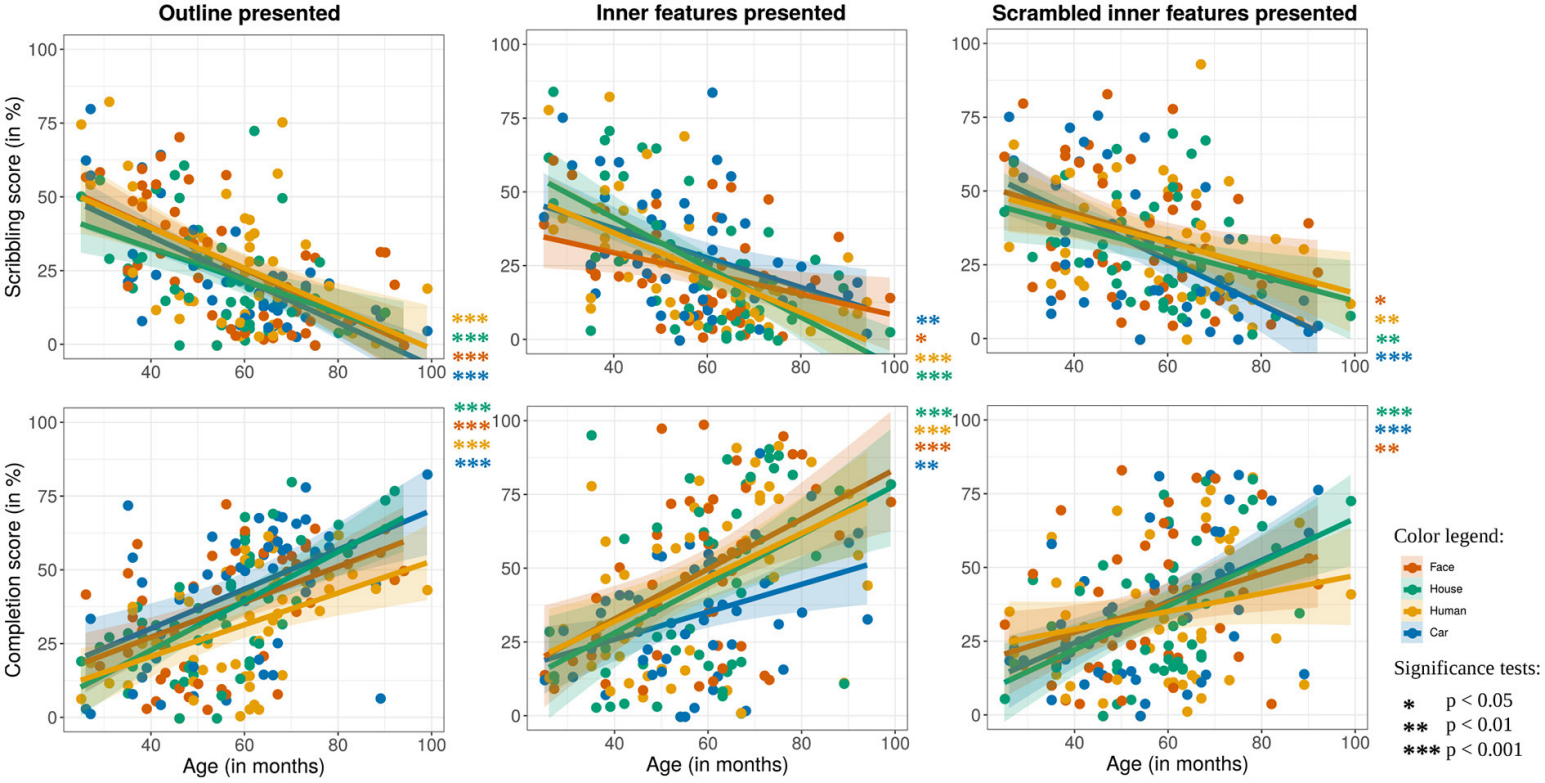
How scribbling **(top)** and completion score **(bottom)** develop across the age of the children, separately displayed for drawings where the outline **(left)**, the inner features **(middle)**, and the scrambled features **(right)** were presented. The colors refer to the category of the presented stimuli. Each point represents the average score of all ratings of one child drawing, and the lines show linear model fits. Reproduced with permission from Philippsen et al. (2020).

First, how the scribbling and completion scores changed with the children's age was tested. It was found that children scribbled less [$\chi_{\left(1\right)}^{2}=56.734,p<0.001$] and completed more [$\chi_{\left(1\right)}^{2}=42.441,p<0.001$] with increasing age, which confirms hypotheses 1 and 2. This trend can be observed in Figure 5 for all stimuli presentation conditions and categories. In these figures, each point corresponds to the average rating that a single drawing of one child received. Regression lines were computed via linear mixed-effect models, using age and AQ as fixed effects, and child and rater identity as well as stimulus type and category as random effects.

Hypothesis 3 stated that children would draw similarly on stimuli in the outline (i) and the inner-feature condition (ii). To test this hypothesis, the effect of stimulus condition was tested on the subset of drawings that were performed in the outline and inner features conditions, using child identity as a random effect. In fact, no main effect could be found of the stimulus presentation condition on scribbling [$\chi_{\left(1\right)}^{2}=0.0008,p=0.98$], tracing [$\chi_{\left(1\right)}^{2}=3.1795,p=0.075$], or coloring [$\chi_{\left(1\right)}^{2}=3.541,p=0.060$]. However, there was a main effect of the condition on completion [$\chi_{\left(1\right)}^{2}=29.542,p<0.001$], which indicates that children's tendency to complete a drawing was affected by the conditions. A possible explanation is that, because of their experience with coloring pictures, the outline condition could have prompted children to fill in the drawings instead of completing them (an overall lower completion score can be observed in Figure 5 for the outline condition).

**Figure 6:**
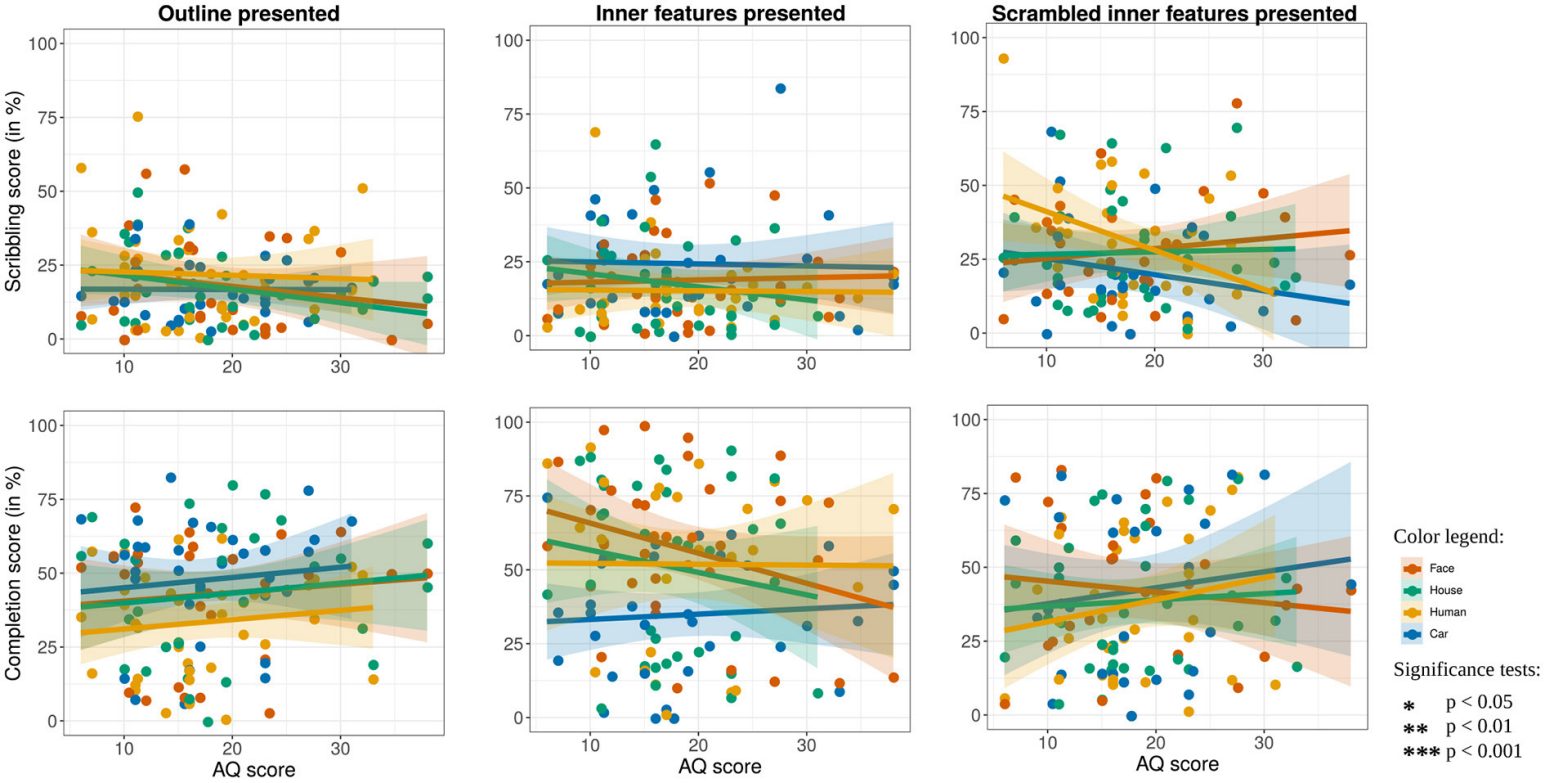
How scribbling **(top)** and completion score **(bottom)** depends on the AQ score of the children, separately displayed for drawings where the outline **(left)**, the inner features **(middle)**, and the scrambled features **(right)** were presented. The colors refer to the category of the presented stimuli. Each point represents the average score of all ratings of one child drawing, and the lines show linear model fits.

Regarding the AQ score, results revealed that the AQ score did not have a significant effect on either scribbling [$\chi_{\left(1\right)}^{2}=0.51,p=0.48$] or completion [$\chi_{\left(1\right)}^{2}=1.10,p=0.23$], rejecting hypothesis 4 (see Figure 6).

Hypothesis 5 stated that children would draw in different ways on the inner features and the scrambled conditions, depending on their AQ scores. However, an interaction effect of stimuli presentation condition with the AQ score on completion was not found [$\chi_{\left(1\right)}^{2}=3.2718,p=0.07$].

In summary, developmental changes in children's drawings could be confirmed (hypotheses 1 and 2), but no systematic relationship between drawing style and AQ score could be established, thus rejecting hypotheses 4 and 5. Whether the outline or the inner features were presented to the children appears to have slightly affected their drawing tendencies (Hypothesis 3). In addition, as Figure 5 shows, the developmental change was less significant in the scrambled inner features condition when compared to the outline or the inner features conditions. In particular, in the scrambled feature condition, less change in the scribbling and completion score with age can be observed in the animate categories (face and human figure). This result could indicate that children had more difficulties in interpreting the scrambled animate figures compared to scrambled inanimate figures, resulting in a lower degree of completion, and a higher degree of scribbling even at older ages. However, it is also possible that the difference in the scribbling score was affected by a lower tendency among raters to rate a scrambled picture as a correct completion.

An exploratory analysis of the other measured drawing styles (tracing of existing lines and coloring of the presented shapes) showed that these styles were present at all ages and neither decreased nor increased significantly with age.

## 4. Results II: Convolutional neural network analysis

In this section, three analyses were conducted. First, an evaluation was performed to assess whether the features extracted by different layers of the CNN appropriately reflected the differences in drawing style observed in the adult rating analysis. This analysis was a verification procedure that ensured that the features extracted by the CNN provide meaningful information about the children's drawings. Second, it was evaluated how children of different ages changed their style of drawing depending on the stimulus presented to them. Finally, the analysis performed for different age groups was conducted for each child, to investigate individual differences that are present in their drawings.

### 4.1. Quantifying children's drawing style

The aim of the first analysis was to verify that the extracted features of the CNN represent differences in the drawings that are meaningful for the analysis of the present study. This analysis was required as the applied CNN has been trained on photographs; thus, it could not be guaranteed that the extracted features could also differentiate abstract images such as line drawings. In particular, correct classification performance cannot be expected due to the fundamentally different nature of the pictures. However, by training on the large ImageNet dataset, it is likely that the network acquired the capability to represent general visual features that are useful for the characterization of drawings, and it has been widely shown that networks trained for one task can be used to solve other tasks (Shin et al., 2016). In fact, similar usage of the network for performing relative comparisons between drawings has been demonstrated successfully in previous research (Long et al., 2018, 2019). It was also shown in Long et al. (2019) that the features extracted in the fully connected layers contained sufficient information for classifying the drawings of older children with regard to the object that they represented.

Here, an examination was done of the feature representations obtained from seven different layers of the network, in line with Long et al. (2018): the five pooling layers and the two fully connected layers. To test whether different drawing styles led to distinct activation of network layers, the results of the rating study was used as a baseline (Section 2.4.1). Specifically, a set of drawings was generated for each of the four drawing styles (scribbling, coloring, tracing, and completion) by using pictures that received an average rating of above 70^3^. This included a total of 19 drawings for scribbling (from children with a mean age of 42 months), 68 drawings for completion (mean age 70 months), 23 drawings for tracing (mean age 55 months), and 93 drawings for coloring (mean age 60 months).

While children showed different drawing styles, adults always completed the drawings. Thus, if the feature representations of the CNN appropriately capture differences between drawing styles, it can be expected that the child drawings that exhibited scribbling, tracing, or coloring would more strongly differ from the adult drawings, when compared to the children's completed drawings. To test this hypothesis, the Pearson distances (Equation 1) between the drawings of the selected sets and adult drawings was measured. Specifically, comparisons were made between the drawings of adults and children that were performed on the same presented stimulus.

**Figure 7:**
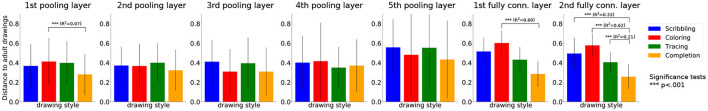
Average distance of drawings that received an average rating of above 70 for a specific drawing style to adult drawings, for four different drawing styles. The distance was computed in either of seven different layers of the CNN. Significant differences are shown which hold for a cut-off of 60, 70, as well as 80.

As Figure 7 shows, a differentiation of drawing styles can be observed in the fully connected layers which represent more global, conceptual features of the drawings. In particular, in the highest network layer, drawings that were rated as completed were significantly more similar to adult drawings than other types of drawing. The highest dissimilarity from adult drawings is found for coloring behavior, followed by scribbling and then tracing behavior.

The distances of drawings with scribbling to adult drawings exhibited a slightly higher variability than for other drawing styles, in line with the qualitative observation that scribbling was the most variable category, ranging from drawings with a few meaningless strokes to scribbles all over the picture. The extracted features, thus, seem to be influenced by general features of the drawing such as the number of strokes and the manner in which these strokes were performed. Nevertheless, a lower distance to adult drawings for completed figures indicate that the extracted features, specifically of the second fully connected layer, sufficiently separate the differences in drawings such that it can be used to quantitatively compare drawings with each other.

### 4.2. Quantifying developmental changes of children's drawings

In contrast to previous studies that used completion tasks (Saito et al., 2014), the designed stimuli set in this study consisted of multiple drawing stimuli, enabling a comparison of how children adapted their way of drawing when the presented stimulus was modified. Following the analysis proposed in Long et al. (2018), it was investigated how children's ability to distinctively draw on different stimuli changed through the course of their development. As a single child drew only on a subset of stimuli, a visualization of how children drew on one stimulus compared to another stimulus required a summarization of the children's drawings into larger groups. Therefore, the drawings were divided into four age groups with (as far as possible) equal distribution of the number of drawings within each group:

- Age group 1: up to 44 months (146 drawings),
- Age group 2: between 45 and 58 months (144 drawings),
- Age group 3: between 59 and 67 months (152 drawings),
- Age group 4: older than 67 months (149 drawings).

The Pearson distance was used to estimate the distances between the drawings. In this analysis, the distances were computed between drawings of children from the same age group on two different stimuli (e.g., the outline of a face and the inner features of a house). To avoid the influence of the presented part of the drawing on this analysis, the presented (black) part was removed before feature extraction. Specifically, for each age group and additionally for the adult drawings, the average feature vectors were determined for each of the 12 stimuli, and subsequently the pairwise differences between the vectors were computed, resulting in a 12× 12 matrix. This matrix is known as the representational dissimilarity matrix (RDM), as defined in Kriegeskorte et al. (2008) and Long et al. (2018), and reflects how the participants adjusted their drawing style when bottom-up perceptual information changed.

**Figure 8:**
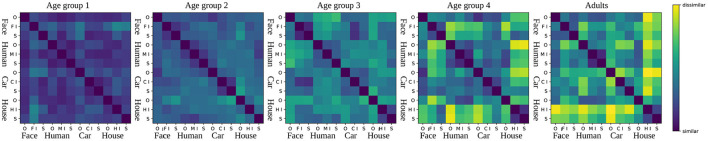
Relative similarity between children's drawings on one stimulus compared to another stimulus for all combinations of stimuli, separated for four different age groups of children as well as for adults. Blue indicates high similarity, and yellow indicates higher dissimilarity. Stimuli are labeled as O for outline, I for inner features, and S for scrambled features. Reproduced with permission from Philippsen et al. (2020).

Figure 8 displays the RDMs for the four age groups and, as a comparison, for adult drawings. The highest network layer was used for feature extraction, as this layer was found to best reflect differences in drawing style in Section 4.1. The matrices show comparisons between all pairs of stimuli sorted by stimulus category. For adults and older children, clusters of similar drawings can be observed close to the diagonal, indicating that these participants flexibly adapted their drawings depending on the category of the presented stimulus. In contrast, younger children perform similar drawings on all presented stimuli.

A statistical analysis of all similarity values of one age group compared to the similarity values of adults showed significant differences between adults and children of age groups 1, 2, and 3 (*p* < 0.001), whereas there was no significant difference between children of age group 4 and adults (*p* = 0.06).

This analysis demonstrated that the ability of children to distinctively adapt their drawings depending on the presented stimuli gradually develops over age.

### 4.3. Quantifying individual differences of children's drawings

A qualitative inspection of the drawings indicated that children's drawings differed significantly not only depending on the child's age, but also between individuals. The analyses showed that AQ scores did not predict children's drawing tendencies; however, there might be other systematic differences in children's drawings that are yet to be discovered. This section deliberates an exploratory analysis that was conducted to test the utility of CNN features to quantify certain aspects of children's drawings. A potential measure is introduced, along with discussions on what it might convey about children's drawings. It is important to note that this analysis remains exploratory, and the objective is not to derive conclusions but to inspire new types of analyses that are yet to be explored.

**Figure 9:**
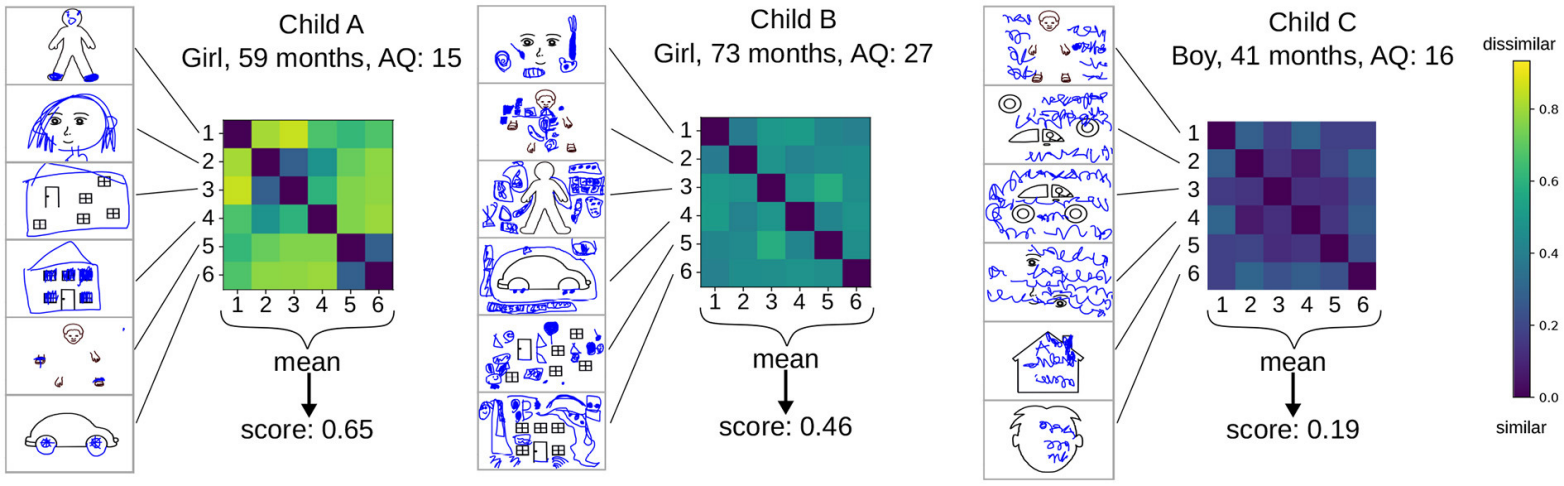
Examples of the RDM matrices individually computed for three children who show qualitatively different styles of drawing.

The main idea is that children's tendencies to rely more strongly either on their prior or on sensory information might be revealed by looking at the RDMs of individual children, as the RDM displays how similarly a child drew on one object, compared to other objects. Thus, the RDM of a child may elucidate whether the child drew similarly on all stimuli or changed the way of drawing depending on the presented, bottom-up stimuli. Specifically, the assumption is that when drawings performed on two different stimuli closely resemble each other, the child only moderately took into account the presented stimuli and instead followed his/her own drawing preferences. In contrast, if children adapted their drawing style depending on the presented stimulus, they might have been more strongly influenced by bottom-up information. Here, this idea was tested by computing the RDMs individually for all children (only the 103 children that saw the complete set of six stimuli were included in this analysis for better comparability). The analysis was performed on the highest layer of the CNN such that the contextual information that the CNN extracts could be included. Figure 9 displays examples of the individual RDM for three children that showed qualitatively different styles of drawing. Child A completed most of the stimuli in the way that was expected, whereas child C scribbled on all the stimuli. This difference can be clearly observed in the differences between the corresponding RDMs: Child A exhibits a higher degree of dissimilarity in the drawings. The mean of the entries of the RDM (excluding the diagonal) reflects this difference^4^. The drawings of child B differ from the drawings of the other two children: although the child produced representational drawings, she did not complete the drawings as expected; instead, she drew objects next to the stimuli that might or might not have been related to the presented stimuli. For this child, the mean of the RDM results is a score that lies between the scores of children A and C. Following the interpretation of this measure, drawings on the stimuli were more diverse than in child C, but were less adapted to the stimuli than in child A. Some readers may agree with this interpretation, while others may disagree. Even though the drawings of child B appeared to be more repetitive compared to child A, they might still be adapted to the presented stimuli, although not in the expected manner. For example, the child might have drawn the monster at the left of the house, intending to show the house's owner. The lower RDM score for child B compared to child C indicates that the CNN is capable of capturing not only the adaptation to the presented stimulus but also the amount of contextual meaning of drawings, to a certain degree. This is an inherent capability of the CNN, as it has been trained with a large number of real-world images. Thus, a score such as the one presented, might have the potential to provide a novel analysis technique for large datasets, based on objective criteria that do not require verbal description of different types of behaviors as necessary for a rating analysis.

**Figure 10:**
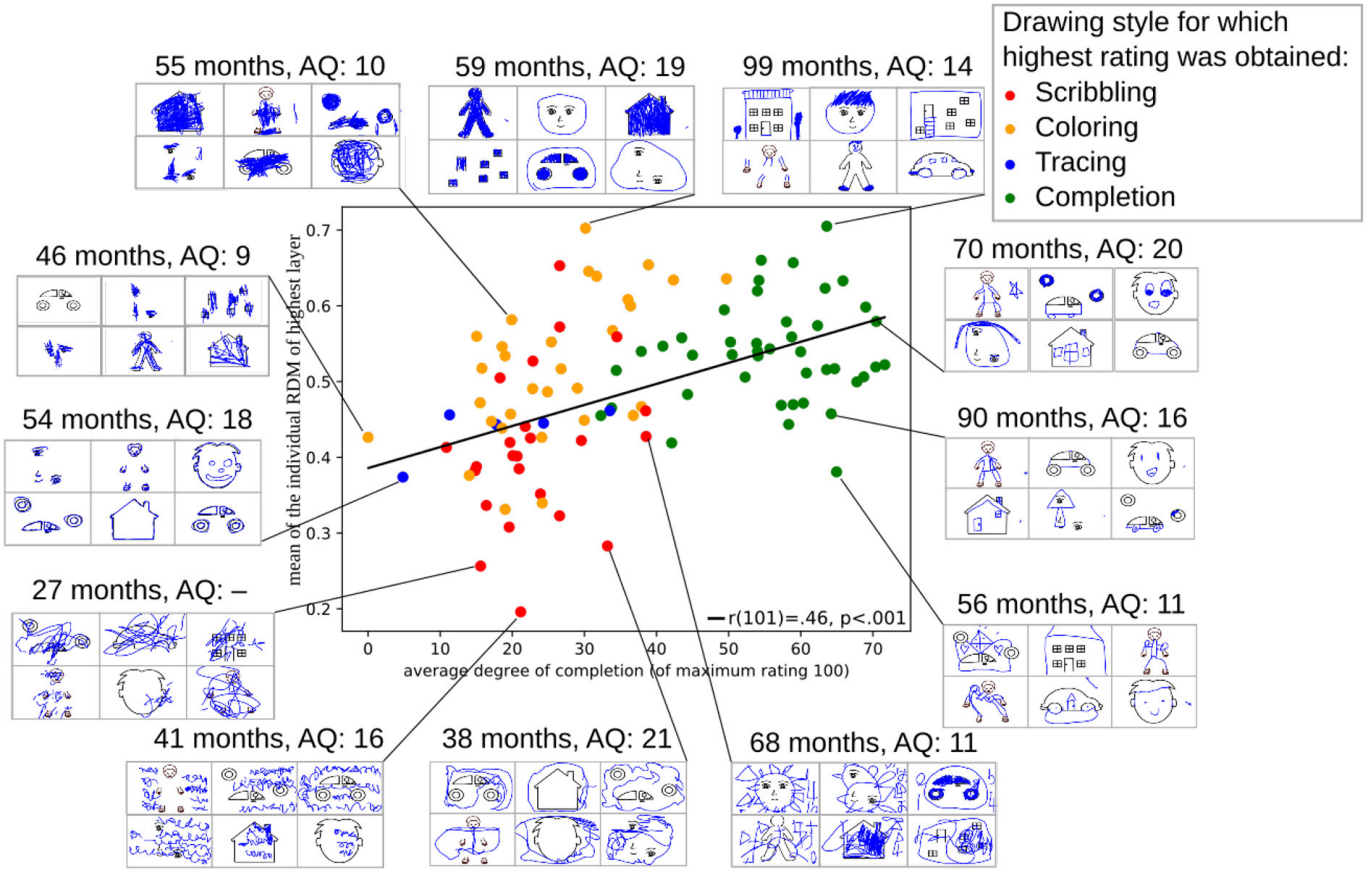
An exemplary score computed from the RDM matrices of all children (y-axis), plotted against the completion score acquired in the rating analysis (x-axis), including some examples of children's drawings. Here, the score is computed as the average of the RDM matrix of the highest layer of the CNN.

To investigate the potential of the proposed measure, the score was plotted against the completion score measured in the rating study, as plotted in Figure 10. Each point in this graph corresponds to the value computed from the drawings of one child, and the colors indicate the most prominent drawing style that the particular child showed (i.e., which drawing style obtained the maximum average rating across all the drawings of the child). An interactive version of the plot where all drawings can be inspected is available via the source code repository ^5^.

The RDM score correlated with the completion score [*r*(101) = 0.46, *p* < 0.001]. Nevertheless, the score reveals additional variability that is not fully explained by the completion score. For example, the 59-month-old child at the top of Figure 10 obtained a high score because she drew differently on the face-related stimuli compared to all the other stimuli. The 55-month-old child on the top left showed only scribbling-like behavior but was nonetheless characterized by a relatively high score as scribbling flexibly adapted to the stimulus position. In contrast, even children who completed the drawings well, such as the 56-month-old child at the bottom right, may achieve a relatively lower score compared to other children who complete the stimuli. It is posited that the lower scores could be attributed to the similarity between the drawings on the two human figure stimuli as well as the generally low degree of contextual information in the drawings. In contrast, the 68-month-old child at the bottom, who mainly showed scribbling tendencies, received a relatively high RDM score since many details were provided in the scribbled drawing. The comparison of the score to the drawings indicates that the score is able to quantify intuitive impressions that observers might have about the child's drawing. Thus, it could be useful as a behavioral measure to efficiently analyze how strongly children consider the presented stimuli while drawing.

However, in its current form, this score has a number of limitations. While contextual information sometimes seemed to be reflected in the score (cf. Figure 9), it cannot be guaranteed in all cases. As the CNN has been trained with naturalistic data, it only accounts for markedly different types of stimuli to a certain degree. Furthermore, the score does not necessarily reflect the children's drawing style but might also be influenced by factors such as the amount of “ink” used and the positions of the drawings, which may negatively affect the interpretability of the score. Further investigations are required to understand the effects of specific drawing manipulations on the RDM score.

Improvements can be made either at the level of task design or at a computational level. For example, modifying the set of stimuli that children are presented with could help improve RDM comparability between children, enabling better interpretation of individual differences, as presented in Figure 10. For example, currently, children may see the same stimulus twice, although in different conditions, which could cause similar drawings that the RDM score does not account for. With respect to score computation, several alternative measures can be imagined. Instead of considering the mean of the entries of the RDMs, additional measures could be computed, such as the highest and lowest values, or the variability. In addition, normalization of the RDMs might be possible based on the visual or semantic similarity of the presented stimuli, to reduce the effect of the presented stimuli on the score computation. Finally, it could be beneficial to use a CNN that is additionally trained on drawing datasets, to improve the capabilities of the network to capture semantic information on line drawings. A full analysis of these possibilities is beyond the scope of this study.

## 5. Discussion and conclusion

In this study, children's representational drawing ability was investigated based on an extended completion task, in which incomplete drawings of four different objects were presented to the children across three different presentation conditions. A two-fold analysis was performed on the collected dataset. First, human ratings collected via a crowdsourcing study provided information on the types of drawing styles. With a larger sample size and diverse stimulus set, the preregistered analysis confirmed developmental changes with age that has been found in previous studies (Saito et al., 2014). However, no consistent effects of AQ scores on children's drawing tendencies have been found.

There might be multiple reasons why the AQ score did not have a measurable effect on the children's drawing tendencies. While we cannot exclude the possibility that changes in drawing behavior by autistic traits might be too subtle and variable to be measurable in a high-level cognitive task such as drawing, also limitations of this study might have contributed to the outcome. Although our sample size was with over a hundred children larger than other studies that evaluated drawing completion in the past (Saito et al., 2014), it is still relatively small, considering the variability of children's drawings. Studies such as Long et al. (2018, 2019) demonstrate that with electronic devices studies can be designed that allow for collecting sample sizes of over 10,000 drawings. Furthermore, a contributing factor might have been that the AQ score of the children in our collected sample did not show much variation. Also further factors might not have shown sufficient variability to be representative. For instance, given that the drawings were collected in a museum, the socioeconomic variability among participants, although not explicitly measured, can also be expected to be relatively low.

It also has to be considered for the interpretation of the results of the present study that children drew with a stylus pen on a tablet PC whereas most traditional drawing studies used paper as a medium. According to a recent study (Kirkorian et al., 2020), drawing on a tablet PC instead of on paper seems to only have minor effects on the quality of children's drawings (mainly at a younger age of 2–3 years, and mainly when using the finger instead of a stylus pen). It is also possible that the brain mechanisms involved in drawing are affected by the change of the medium. Further research is required to be able to answer this question.

In a second analysis, we adopted the methodology of Long et al. (2018) that is based on a pretrained deep CNN. This analysis allowed an examination of the drawings independent of human perception. Specifically, drawings were evaluated at the level of local or global features. The results revealed that the way children adapt to the presented stimuli becomes more similar to adults with increasing age, which was particularly observed when using features of the higher network layer. The findings demonstrate that the methodology proposed in Long et al. (2018, 2019) for analyzing drawings that children made of specific objects can also be transferred to systematically investigate children's drawing tendencies in a completion task.

Finally, it is proposed that the RDMs of individual children might be useful for investigating individual differences in children's drawings with respect to the predictive coding theory. We explored this possibility by qualitatively examining the differences in children's drawings based on a score computed from the children's RDMs, along with identifying and discussing strengths as well as shortcomings of the measure. The initial analysis showed that the score reflects observable differences in children's drawings and could be relevant in the context of predictive coding, as it indicates the degree to which individual children rely on sensory information compared to their own predictions. However, the current form of the computed score has a number of shortcomings that limit its applicability which need to be addressed in future studies. Even so, we suggest that this style of analysis combined with the task design used in the present research may be useful for studying individual differences in the future, and for quantitatively analyzing drawing tendencies across large datasets.

## Data availability statement

The datasets presented in this study can be found in online repositories. The names of the repository/repositories and accession number(s) can be found at: https://github.com/aphilippsen/MiraikanDrawing.

## Ethics statement

The studies involving human participants were reviewed and approved by Office for Life Science Research Ethics and Safety (The University of Tokyo). Written informed consent to participate in this study was provided by the participants' legal guardian/next of kin. Written informed consent was obtained from the minor(s)' legal guardian/next of kin for the publication of any potentially identifiable images or data included in this article.

## Author contributions

YN, ST, and AP conceived the experiments and analyzed and interpreted the results. AP and ST conducted the psychological experiments. AP implemented and conducted the computational experiments and drafted the manuscript. YN and ST reviewed the manuscript. All authors read and approved the final manuscript.

## Funding

This work was supported by JST CREST Cognitive Mirroring, Japan (Grant Number: JPMJCR16E2), the JST CREST Cognitive Feelings, Japan (Grant Number: JPMJCR21P4), the JSPS KAKENHI (Grant Number: 21H05053), the Institute for AI and Beyond, the University of Tokyo, Japan, and by the World Premier International Research Center Initiative (WPI), MEXT, Japan.

## Conflict of interest

The authors declare that the research was conducted in the absence of any commercial or financial relationships that could be construed as a potential conflict of interest.

## Publisher's note

All claims expressed in this article are solely those of the authors and do not necessarily represent those of their affiliated organizations, or those of the publisher, the editors and the reviewers. Any product that may be evaluated in this article, or claim that may be made by its manufacturer, is not guaranteed or endorsed by the publisher.

## Footnotes

^1^There were nine 2-year-old children, twenty 3-year-olds, twenty-four 4-year-olds, thirty-two 5-year-olds, thirteen 6-year-olds, five 7-year-olds, and one 8-year-old.

^2^OSF preregistration: https://osf.io/tzwkv.

^3^With this value, drawings can be assumed to be typical examples. To demonstrate that the exact choice of this percentage was insignificant, the results for 60 and 80 were also analyzed. In Figure 7, only the significance values with *p* < 0.001 that were found in all three analyses are reported.

^4^Note that a direct comparison of the matrices is not possible because the children drew on different subsets of stimuli.

^5^ https://github.com/aphilippsen/MiraikanDrawing
